# Sensitivity of source apportionment predicted by a Bayesian tracer mixing model to the inclusion of a sediment connectivity index as an informative prior: Illustration using the Kharka catchment (Nepal)

**DOI:** 10.1016/j.scitotenv.2020.136703

**Published:** 2020-04-15

**Authors:** Hari Ram Upadhayay, Sushil Lamichhane, Roshan Man Bajracharya, Wim Cornelis, Adrian L. Collins, Pascal Boeckx

**Affiliations:** a Sustainable Agriculture Sciences, Rothamsted Research, North Wyke, Okehampton EX20 2SB, UK; b School of Environmental and Rural Science, University of New England, Armidale, Australia; c Soil Science Division, Nepal Agricultural Research Council, Nepal; d Department of Environmental Science and Engineering, Kathmandu University, Nepal; e Soil Physics (SoPHY), Ghent University, Coupure Links 653, 9000 Gent, Belgium; f Isotope Bioscience laboratory-ISOFYS, Ghent University, Coupure Links 653, 9000 Gent, Belgium

**Keywords:** Sediment source apportionment, Compound-specific stable isotope (CSSI), Prior information, Sediment connectivity index, Water erosion

## Abstract

Long-chain saturated fatty acid (LCSFA) isotopic composition in tandem with Bayesian isotope mixing models (BIMM) can provide insight into land use-based sediment sources in catchment systems. Apportioning sediment sources robustly, however, requires careful consideration of how additional factors including topography, surface cover and land use practices interact to influence contributions from individual sources. Prior knowledge can be used in BIMM; however, the full capacity of this functionality has not been thoroughly exploited yet in conjunction with sediment fingerprinting. In response, we propose an approach for applying a state-of-the-art BIMM incorporating a sediment connectivity index (SCI) as an informative prior for sediment source apportionment in a highly hydrodynamic catchment in Nepal. A library of LCSFA carbon isotopic composition was constructed for surface soils collected from mixed forest, upland and lowland terraces in the Kharka micro-catchment. δ^13^C values of LCSFA of time-integrated suspended bulk (<2 mm) sediment were depleted by 4‰ compared to the fine (<0.063 mm) sediment fraction. Conventional source apportionment for fine sediment samples without the SCI informative prior suggested that 66% of the sediment is derived from forest soils followed by lowland (19%) and upland (15%) terraces. Incorporation of the SCI as an informative prior in BIMM, however, modified the original source apportionment estimates to 90%, 9% and 1% respectively. The lower contributions from agricultural terraces are explained by landscape complexity comprising small levelled terraces that reduce hillslope-to-channel sediment connectivity. This study demonstrates the sensitivity of BIMM posterior distributions to incorporation of an informative prior based on a SCI. Inclusion of SCI linked to land use and management can provide a more physically-grounded approach to estimating sediment source contributions from biogeochemical tracers, and critically one which generates results better reflecting what makes good environmental sense in the context of land management and visual evidence of sediment mobilisation and delivery.

## Highlights

• Sensitivity of a BIMM to connectivity indices was tested.
• Community forest contribution to fine sediment increased 23% with informative priors.
• δ^13^C-FAs values must be obtained from the same size fractions for both source soil and target sediment.
• Topographic features and forest management highly influence sediment generation and delivery.

## Graphical abstract

**Figure:**
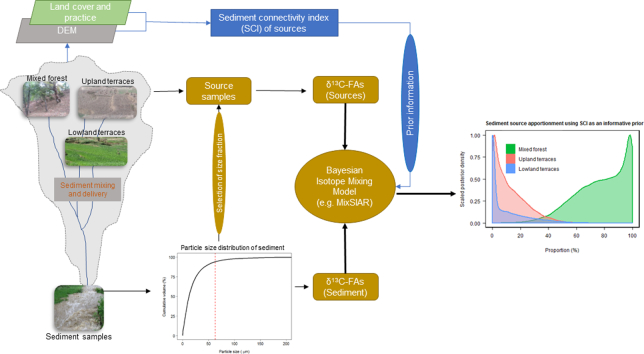

## 1 Introduction

Accelerated soil erosion by water on hillslopes is a global environmental problem leading to loss of soil fertility and reduced agricultural productivity, degrading freshwater quality and increasing lake and reservoir siltation (Montanarella et al., 2016). Soil erosion is a complex and dynamic process due to the non-linear interaction of climate, topography and land use at the catchment scale (Garcia-Ruiz et al., 2015; Verheijen et al., 2009) and involves soil detachment, hillslope sediment delivery and fluvial transfer and deposition processes. Human activities have, however, altered the magnitude of these processes through inappropriate agriculture and forest management, construction works, mining and climate change. All of these activities have a propensity to increase soil erosion risk and the efficiency of hydrological transfers of sediment from hillslopes to river channels. In particular, mountainous headwater catchments respond very quickly to effective rainfall due to their short and steep nature which results in a decrease in transit times for mobilised sediment and associated pollutants (Hrachowitz et al., 2016; McDonnell and Beven, 2014). Minimising water erosion and reducing sediment fluxes from headwater catchments is therefore critical for maintaining healthy aquatic ecosystems downstream. Here, reliable information on the proportional contributions of fine-grained (<0.063 mm) sediment from different land uses is a key requirement for targeted soil erosion control and sediment management.

Particle size distribution (PSD) is one of the most fundamental physical attributes influencing soil erosion and the content of nutrients and contaminants in eroded materials (Laceby et al., 2017). Land use and soil surface properties exert a strong control on PSD by helping or hindering soil detachment, i.e. erosive risk and subsequent sediment mobilisation at the catchment scale (Ding and Huang, 2017; Wang et al., 2008). Soil erosion, mobilisation and delivery processes generally result in the fining of sediments due to preferential mobilisation and transport of fine and light soil particles. Coarse fractions are trapped on hillslopes, and only finer fractions move down the hillslope (Silburn et al., 2011). In addition to hydrodynamic sorting, breakage and abrasion of sediment particles during downstream transport in high-energy mountainous catchments can also result in the fining of particle size. Particle size selectivity due to erosion and fluvial processes not only reduces particle size but also results in potential differences in biogeochemical tracer properties and concentrations in sediments relative to their sources (Laceby et al., 2017). Consequently, fine-grained sediment resulting from soil erosion and sediment transport is considered one of the most important causes of aquatic ecosystem degradation worldwide due to its role in the transfer and fate of nutrients, trace and heavy metals, radionuclides and emerging micropollutants (e.g. pharmaceuticals and pesticides) (Bilotta and Brazier, 2008).

Sediment fingerprinting techniques are increasingly used to apportion the sources of downstream sediment using a wide array of physical and chemical tracers (Collins et al., 2017; Owens et al., 2016; Tang et al., 2019). Of the various tracers (e.g., biogeochemical, optical and isotopic properties) of sediment used to date, the use of Long-chain saturated fatty acids (LCSFAs, carbon chain length >20 carbon atoms) as specific isotopic tracers appears promising for apportioning land use contributions to fine-grained sediment at the catchment scale (Reiffarth et al., 2016; Upadhayay et al., 2017). Soils under different vegetation cover (i.e., land use) can potentially be distinguished by the analysis of the carbon isotopic composition of LCSFAs since stable carbon (δ^13^C) isotopic values record vegetation and land use information. Moreover, LCSFAs are associated with mineral matter in soils due to their greater hydrophobicity, thereby rendering them more resistant to degradation, while increasing their propensity to move with sediment. Sediment fingerprinting relies upon the characterisation and comparison of the compound specific stable isotope (CSSI) values of soil from potential sources within a catchment and of target sediment sampled at the point of interest (Upadhayay et al., 2017). Previous studies in CSSI sediment fingerprinting (e.g. Alewell et al., 2016; Gibbs, 2008; Upadhayay et al., 2018b) focused on bulk (<2 mm) soil and sediment samples for the extraction of FAs and their carbon isotopic analyses. This approach, however, is insensitive for accounting for the impact of soil erosion processes on LCSFA contents due to particle size selectivity. The association and stabilisation of LCSFAs increases with decreasing particle sizes due to the strongest binding of lipids on mineral surfaces or organo-mineral complexes (Griepentrog et al., 2015; Wiesenberg et al., 2010). Consequently, many studies have reported enrichment of free FAs derived from plant lipids in the clay and silt sized fractions in soil (Jansen and Wiesenberg, 2017; Quénéa et al., 2004; Quenea et al., 2006) and in target sediment (Yu et al., 2019). Moreover, mineral protection of lipids also depends on the cultivation pattern (Li et al., 2018). Collectively, these studies clearly showed that LCSFA content is sensitive to changes in PSD and land use. To address this issue, the δ^13^C value of LCSFAs should be obtained from the same size fractions for both source soil and target sediment samples to ensure direct comparability.

Identifying the sources of the fine-grained sediment remains challenging in complex landscapes (Tang et al., 2019; Upadhayay et al., 2018b) meaning that innovative approaches continue to be needed to help elucidate how catchment configuration, surface cover and land use practices interact to influence hillslope-to-channel sediment delivery from each source. Landscape gradient, ground cover and farming practice strongly govern hillslope sediment connectivity at the catchment scale (Fryirs, 2013). However, linking these factors (e.g. landscape gradient, farming practices, etc.) remains an important challenge in sediment fingerprinting since spatially diverse landscapes have a propensity to buffer and disconnect sediment pathways. Applications of BIMM to fingerprint sediment sources are steadily increasing (Blake et al., 2018; Upadhayay et al., 2017) and such tools permit incorporation of informative priors for sediment proportions from each source type at the catchment outlet. Informative priors represent prior knowledge and assumptions for the proportional contributions of sources in the study catchment that can be updated by measured or derived data to help estimate source contributions. Because of ambiguity surrounding prior choice, informative priors currently remain underutilised in sediment source apportionment, despite the growing global uptake and application of the fingerprinting approach. Prior knowledge of the typical relative contributions from e.g. channel bank sources has been used to constrain solutions generated by both frequentist (Collins et al., 2010) and Bayesian (Blake et al., 2018) approaches. Independently, Thompson et al. (2013) reported the utility of structural connectivity (i.e., connectivity potential determined by the distribution of landscape features) to predict high-risk areas for sediment within catchments. Sediment connectivity is the degree to which eroded materials are connected and interact with water flow and is considered a primary control for the delivery of sediment from slopes to downstream aquatic receptors (Leibowitz et al., 2018). However, sediment fingerprinting approaches, to date, typically assume equal connectivity of eroded materials with transport pathways among all sources under scrutiny even though such connectivity frequently differs among sources due to the presence or absence of sediment buffers, barriers and blankets. Many other factors including rainfall, topography and the spatial distribution of different land uses can also affect sediment connectivity. Such connectivity can further be altered by human impact, often in a complex manner and varying over both time and space (Heckmann et al., 2018; Leibowitz et al., 2018). It would therefore seem logical that use of a sediment connectivity index (SCI) for each land use as an informative prior in BIMM could be promising for taking explicit account of a ground-based physical factor controlling slope-to-channel sediment delivery and concomitant source proportions.

Against the above context and since the crucial role of sediment connectivity is implicit, rather than explicit, in source fingerprinting studies to date, the overall aim of this study was to assess the implications of incorporating a hillslope-to-channel SCI as a potentially explicit informative prior in a BIMM. In doing so, we assessed the sensitivity of the predicted source proportions from the BIMM to the incorporation of a SCI based on either physical connectivity alone, or a combination of physical connectivity and land cover and management. Because sediment contributions from sources have been previously shown to be dependent on the sediment source-sink relationships comprising the landscape sediment cascade system, we hypothesised that sediment source contributions at the study catchment outlet would be dominated by the source that has a higher sediment connectivity index. We therefore compared the uninformative posterior distributions generated by the conventional reference BIMM with alternative posterior distributions based on two versions of the SCI. Here, the lack of a statistically significant difference between the uninformative and either set of informative posteriors would suggest that the inclusion of a prior based on a SCI is not informative (Van Dongen, 2006). Alternatively, sensitivity in the posteriors, as indicated by progressive change in conjunction with the inclusion of either of the two versions of the SCI in the BIMM, would suggest that SCI is indeed informative and worthy of inclusion (Lemoine, 2019), especially in the context of making better environmental sense on the basis of visual observations of land management, erosion and sediment delivery in the study area (Slattery et al., 1995; Slattery et al., 2000; Walden et al., 1997).

## 2 Methodology

For comprehensive reviews of sediment source fingerprinting and associated methodologies in general, readers are referred to Owens et al. (2016) and Collins et al. (2017). For reviews specifically more focussed on using Bayesian approaches with sediment source fingerprinting, readers are referred to Upadhayay et al. (2017) and Davies et al. (2018) and on issues of particle size effects on sediment fingerprinting, readers are referred to Laceby et al. (2017).

### 2.1 Study catchment

**Fig. 1:**
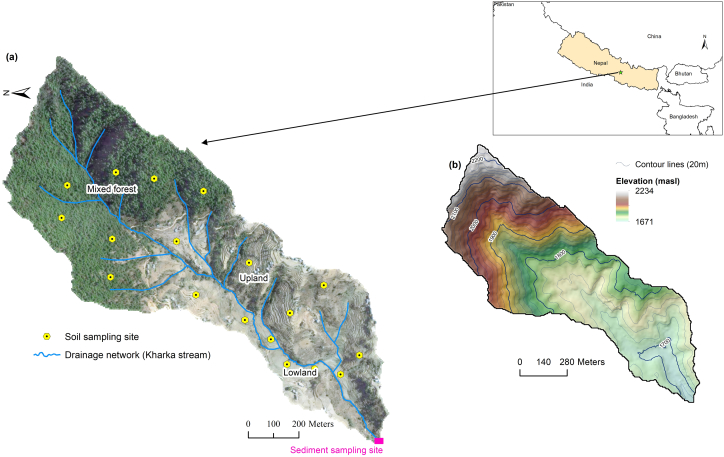
Location of the Kharka catchment in Nepal (inset) with distribution of (a) land use as well drainage network and (b) elevation.

**Fig. 2:**
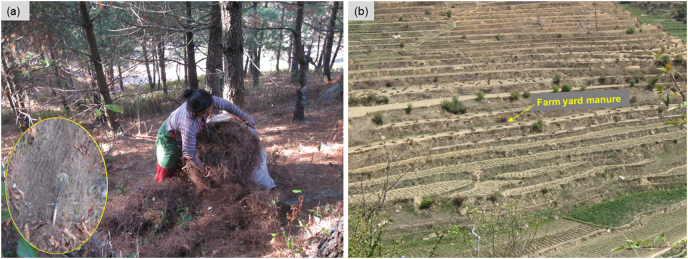
Surface disturbance (inset) due to litter collection using a hand hoe in the (a) community forest and application of forest litter-based farmyard manure on the (b) agricultural terraces in the Kharka catchment.

The Kharka (1 km^2^) is one of several sub-catchments of the Kulekhani River basin, Nepal, which drain into the Indrasarobar reservoir. The Kharka stream flows south-westerly until its confluence with Chitlang stream (Fig. 1a). Elevation ranges from 1671 to 2234 m a.s.l and slope inclination reaches up to 65° (approximate average 29°), especially in the northern portions of the study area (Fig. 1b). Consequently, the catchment has limited floodplain development which results in fewer opportunities for temporary storage of fine-grained sediment. The climate is characterised by high rainfall (~1750 mm yr^−^^1^) with two distinct seasons, i.e., a summer monsoon and dry winter. About 77% of the rainfall occurs during the monsoon season (June–September) with only a small fraction i.e. 23% during the non-monsoon season (DHM, 2015; Upadhayay et al., 2018b). The overlying soil is classified as Cambisols (Dijkshoorn and Huting, 2009). The main land uses comprise mixed (deciduous and pine trees) forest (MF; 46%) and cultivated land including both upland (UP; 44%) and lowland (LL; 10%) terraces with areas of housing (Fig. 1a). The forest is managed by the community and is a vital resource for livelihoods due to the collection of leaf litter (Fig. 2a) for animal bedding and compost and for fodder for livestock consumption. Terracing is a dominant feature of agricultural land use (Figs. 1a, 2b). Maize may be grown as a monoculture crop on upland terraces or mixed and inter-cropped with vegetables (potato, cabbage, cauliflower, legumes). Farmers practise flood-irrigation on the lowland flat terraces using stream water conducted through small channels during the monsoon season. Farming of wheat and commercial vegetables is dominant during the winter season on lowland terraces. Cultivated land receives inorganic fertilizer as well as farmyard manure (FYM; Fig. 2b) as well as chicken litter.

### 2.2 Soil and sediment sampling

A reconnaissance survey identified three primary potential sediment sources in the Kharka catchment, i.e., mixed forest, upland and lowland terraces. From each source type, topsoil (3 cm) composite samples (each drawn out of a pool of 15 random subsamples of almost equal amount collected using the same corer) from different geographic locations (Fig. 1a) within the catchment were obtained using stainless steel corer with internal diameter of 7.5 cm. The use of composite samples to reduce sample numbers and associated analytical costs is standard in source tracing procedures, but there are potential trade-offs in terms of the results (e.g. reduced information on tracer variability) generated on this basis (Collins et al., 2017). Subsampling and compositing addresses small scale random spatial variability in tracers (Collins et al., 2019). Two time-integrated mass flux samplers were deployed at the catchment outlet (Fig. 1a) from April to November 2015, with sediment samples being retrieved in June, August and November to characterise the temporal variability of fine-grained sediment fluxes (Phillips et al., 2000). Soil and sediment samples were air dried for 20–25 days in polytunnel, gently disaggregated and dry sieved through a 2 mm mesh for further processing.

### 2.3 Particle size separation and extraction of free FAs

Dried and ground soil and sediment (2 g) was used for particle size analysis. Briefly, organic matter was oxidised by H_2_O_2_ and the sample dispersed using 5 ml of 5% sodium hexametaphosphate. Particle-size distributions were measured using laser diffraction (Mastersizer 2000, Malvern) in the range of 0.00002 to 2 mm. Each sample was measured 5 times and the corresponding mean values calculated (see Table S1). Clay (<0.002 mm) and silt (0.02–0.063 mm) percentages in the source soil and target sediment samples were estimated based on the particle size distributions. The D_90_ of the sediment samples (Fig. S1) was used to inform further sieving of the source samples to reduce the influence of particle-size effects on fatty acids content (Quénéa et al., 2004). 100 g of the soil or sediment samples (<2 mm) were weighed in 500 ml bottles and visible organic matter particles were removed manually. The samples were dispersed (1:5 soil:water) with cooled distilled water (24 °C) by shaking at 300 rpm for 1 h. The coarse and fine sand fraction (>0.063 mm) were then separated from the silts and clays by wet-sieving (mesh size 0.063 mm) with 500 ml of cooled distilled water. After the fractionation, samples were dried at 45 °C in an aluminium tray in an air-fan oven. The <2 mm and <0.063 mm fractions hereafter are referred to as the bulk and fine fractions, respectively.

### 2.4 FAs extraction and compound-specific stable isotope analysis

Free FAs were extracted from the bulk and fine fractions of the source soil and sediment samples using accelerated solvent extraction (ASE 350 Dionex) with dichloromethane (DCM): MeOH (9:1 v/v). Further details for the FA extraction and purification and analysis are reported in Upadhayay et al. (2018b). To determine the δ^13^C values of FAs (δ^13^C-FAs), the contribution of the δ^13^C values of the added methyl group was subtracted from the δ^13^C value of the analysed FAME, resulting in a corresponding uncertainty on the Vienna PeeDee Belemnite (VPDB) scale of <0.6‰. Only the FAMEs of long-chain even carbon numbered FAs (C_20_ – C_32_) were used for further analysis since their isotopic composition is less likely affected by decomposition and other processes (Reiffarth et al., 2016; Upadhayay et al., 2017).

### 2.5 Estimation of the SCI as prior information

**Fig. 3:**
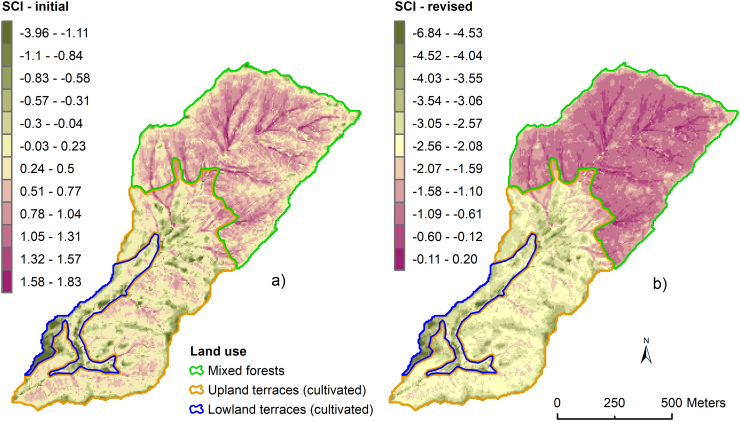
Sediment connectivity index (SCI) of each land use estimated weighing by surface roughness index (a) SCI-initial and with land cover and practice (b) SCI-revised.

The sediment connectivity index (SCI) score for each sediment source was calculated to evaluate the degree to which each land use facilitates the hillslope-to-channel transfer of water and sediment. Here the GIS-based approach developed by Cavalli et al. (2013) and based on a study reported by Borselli et al. (2008) was used, viz.:

(1) $$\mathrm{SCI}=\log_{10}\left(\frac{D_{\mathrm{up}}}{D_{\mathrm{dn}}}\right)=\log_{10}\left(\frac{\bar{W}\bar{S}\sqrt{A}}{\sum_{i}\frac{d_{i}}{W_{i}S_{i}}}\right)$$

where W_i_ is the weighing factor for the ith cell (dimensionless), $\bar{W}$ is the average weighting factor for the upslope contributing areas (dimensionless), S_i_ is the slope gradient of the ith cell (m m^−^^1^), $\bar{S}$ is the average slope gradient for the upslope contributing areas (D_up_) (m m^−^^1^), A is the upslope contributing (D_dn_) areas (m^2^) and d_i_ is the length of the ith cell along the downslope path to the sink (m). SCI is the sediment connectivity index, the value of which is defined in the range of −∞ to +∞, with connectivity increasing for higher SCI values. The weighting factor represents the impedance of sediment flux and runoff due to vegetation, soil and land use management (Borselli et al., 2008; Cavalli et al., 2013). In this study, weighting factors were estimated using 1) a surface roughness index (Cavalli et al., 2013) and, 2) land cover and practices, to estimate SCI-initial and SCI-revised, respectively. Canopy cover, ground surface coverage and practice variations can have a significant impact on the impedance of water and sediment connectivity in mountainous catchments (Llena et al., 2019; Gardner and Gerrard, 2002). Ground cover reduces soil detachment by mitigating raindrop impact and has been shown to be more effective than tree canopy in reducing soil erosion in forest areas (Gardner and Gerrard, 2002; Miyata et al., 2009). A 5 m resolution digital elevation model (DEM; Fig. 1b) obtained from the Advanced Land Observing Satellite World 3D (AW3D) (AW3D, 2017) from the Japan Aerospace Exploration Agency (JAXA) was used after pit removal to estimate the SCI using topographic roughness as weighting factor (SCI-initial; Fig. 3a). SCI-revised (Fig. 3b) scores were obtained after weighting by the RUSLE land cover (C) and practice (P) factors obtained from Renard et al. (1997). Because of the patchy cropping pattern (sometimes each terrace might have very different crops) and land use other than cropping dominates (e.g. fodder trees on the terrace riser), the C factor is normally assigned based on the simple assessment of vegetation cover in the mid-hills, rather than higher resolution analysis of agricultural cropping patterns (Uddin et al., 2016). The terraces in this catchment are so narrow that a digital elevation model of 5 m resolution is unable to capture the width of terraces realistically. Recognising the existence of bench terraces in cultivated areas and absence of them in forest areas, practice (P) was included in the estimation of the weighting factor (see detail in Table S2). Here, the overall weighting scores for forest (0.12) and agricultural land (0.03) were estimated based on the canopy cover sub-factor, surface cover sub-factor and management practice. Finally, average SCI-initial and SCI- revised values were calculated for each land use type (Fig. S2) to incorporate and compare as prior information in the source apportionment modelling using the BIMM.

### 2.6 Data processing using MixSIAR

Individual FA contents and δ^13^C-FA values in the bulk and fine fractions of the source and target sediment samples were compared by *t*-tests (*p* < 0.05). δ^13^C-FA values measured for the individual land use sources were compared using a one-way ANOVA followed by Tukey's honestly significant differences to determine the ability of the individual biomarker tracers to differentiate MF, UP and LL using each grain size fraction. Tests for tracer normality were conducted using an Anderson-Darling test, while homogeneity of variance was assessed using the Bartlett's test. A state-of-the-art BIMM implemented as an open source R package, i.e. MixSIAR, (Stock and Semmens, 2013) was applied for estimating sediment source apportionment. MixSIAR estimates the proportion of each source in the target sediment samples, while accounting for concentration dependence, variability in source isotopic values and various effects (random, fixed and continuous) on the variability of sediment isotopic composition (Stock et al., 2018; Upadhayay et al., 2017; Upadhayay et al., 2018b). Prior to the unmixing process in MixSIAR, the assumption that sediment signatures must be within a polygon bounding the source signatures must be tested. We tested this assumption by transforming an ellipse made from source isotopic tracer data into a perfect circle and projecting the target sediment isotopic data into the same circle (Jackson, 2016). The test provides results in a binary form. This test was satisfied by 3 tracers (C20, C28 and C30) for the bulk and 5 tracers (C20, C22, C24, C28 and C32) for the fine sediment samples (see Fig. S3 in Supplementary information). Subsequently, concentration-dependent MixSIAR (Upadhayay et al., 2018a) was formulated with these selected FAs, using a residual error term (Stock et al., 2018; Upadhayay et al., 2017) and both with and without using the two versions of the SCI (SCI-initial and SCI-revised) as informative priors. Average SCI-initial (MF = 0.634, UP = 0.198, LL = −0.317) and SCI-revised (MF = −0.956, UP = −2.508, LL = −3.126) values for each land use (Fig. S2) were used as prior information during the formulation of the MixSIAR framework. Although including average SCI-initial or SCI-revised values as informative priors for each land use inevitably lost some information that can be gained from these indices, it is important to acknowledge that the current version of the MixSIAR framework does not provide users with the functionality to incorporate the variance of informative priors. For more detail on the use of Bayesian priors and the MixSIAR framework, readers are referred to Stock et al. (2018). The Markov Chain Monte Carlo (MCMC) parameters in MixSIAR were set as follows: number of chains = 3, chain length = 3,000,000, burn = 1,500,000, thin =500. Convergence of model runs was checked via Gelman-Rubin and Geweke diagnostic statistics and these were found to be sufficient (Gelman-Rubin statistic <1.05 and for Geweke, low absolute z-score) (Stock and Semmens, 2013). The best model fit was determined by the deviance information criterion (DIC). To describe sediment source contributions and corresponding uncertainty, means and the 95% Bayesian credible intervals (CI) were reported from the posterior distributions computed for sediment source contributions. Matrix plots (generated by MixSIAR) with correlation values between estimated source proportion indicate the quality of source discrimination by the tracers and assist in evaluating the influence of informative priors in the original model i.e. with unformative prior. Strong correlation among sources can increase the level of uncertainty in model output.

Ideally, the posteriors generated by the different versions of the BIMM would be evaluated using independent data, but such data are rarely available and indeed are often the driver for application of source tracing procedures which provide an alternative to resource demanding conventional direct catchment monitoring methods. In this context, and to provide some evaluation of the influence of the SCI priors, we monitored the bias, accuracy and precision of the informative posteriors for each model relative to the corresponding estimates for uninformative posteriors generated using the conventional BIMM assuming equal connectivity for all sediment sources. Using the posteriors generated by the conventional BIMM with uninformative priors (P_ukt_) and the updated BIMM with either version (SCI-initial or SCI-revised) of the informative priors (P_ikt_), we calculated the bias of each proportional estimate (Derbridge et al., 2015) as:

(2) $$\mathrm{bias}\;\left(p_{\mathrm{ik}}\right)=\left[\left(\sum_{t=1}^{n}p_{\mathrm{ikt}}-p_{\mathrm{ukt}}\right)\times \frac{1}{n}\right]$$

where bias value close to zero represents unbiased estimation with incorporation of informative priors. We further calculated and compared the Monte Carlo variance (i.e., precision) of the posteriors generated using P_ukt_ and P_ikt_ as:

(3) $$\mathrm{var}\;\left(p_{\mathrm{ik}}\right)=\left[\sum_{t=1}^{n}{\left(p_{\mathrm{ikt}}-\frac{1}{n}\sum_{t=1}^{n}p_{\mathrm{ikt}}\right)}^{2}\right]\times \frac{1}{n-1}$$

where a small variance estimate represents increased precision.

Finally, accuracy as the mean square error (MSE) was calculated using bias and variance as:

(4) $$\mathrm{MSE}\;\left(p_{\mathrm{ik}}\right)=\mathrm{var}\left(p_{\mathrm{ik}}\right)+{\left(\mathrm{bias}\left(p_{\mathrm{ik}}\right)\right)}^{2}$$

where smaller values of MSE represent higher accuracy.

## 3 Results

### 3.1 The particle size characteristics of source and target sediment samples

The average cumulative particle size distribution curves for the source and target sediment samples (see Fig. S1, Table S1 in Supplementary information) showed that sediment and lowland source soil samples were predominantly composed of clay (64% and 65%, respectively) and silt (26% and 29%, respectively) with a unimodal size distribution (D_90_ = 0.064 and 0.066 mm for the sediment and lowland source soil samples, respectively). The upland and forest source soils were also predominantly composed of clay (57% and 47%) and silt (27% and 28%, respectively), but with an additional sandy component (D_90_ = 0.082 and 0.120 mm for upland and mixed forest source samples, respectively).

### 3.2 Distribution of FA isotopic composition in source and target sediment samples

Long-chain FA distribution patterns in the source soil and target sediment samples were characterised by a predominance of saturated even-numbered FAs. The dominant FA found in the fine fraction samples of all sources was C30, whereas C24 dominated in the bulk fraction of the soil samples collected from MF and UP (see Fig. S4 in Supplementary information). Both size (bulk and fine) fractions of the suspended sediment samples were dominated by C24. The content of individual FAs was approximately 1.5 times higher in the bulk compared with the finer fraction of the samples, irrespective of those samples being either source soil or target sediment (*p* < 0.05).

**Fig. 4:**
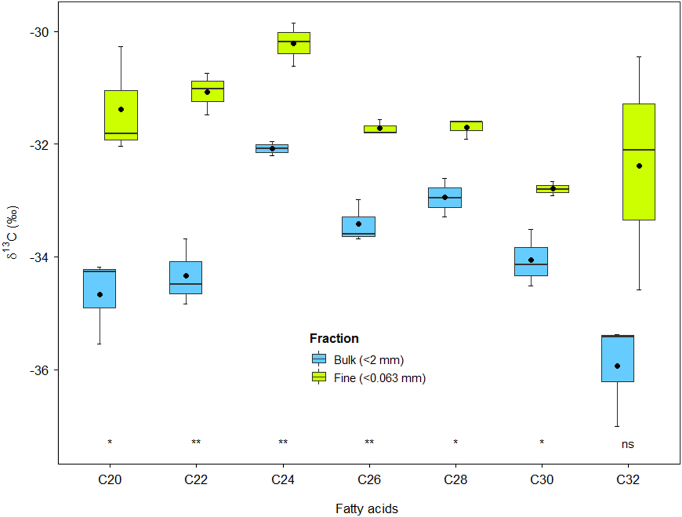
Distribution of isotopic composition of long-chain FAs in the bulk and fine fractions of the time-integrated suspended sediment samples. Asterisks indicate significant differences (ns, *p* > 0.05; *p < 0.05; **p < 0.01) between fractions of FAs. Horizontal line and closed circle in the box indicate median and mean values respectively. The bottom and top of the box refer to the 25th and 75th percentile i.e. interquartile ranges (IQR) and whiskers represent the smallest and largest values within 1.5 times of the IQR.

The stable carbon isotopic composition of long-chain saturated FAs associated with the two particle size fractions of the source soil and suspended sediment samples varied considerably (Table 1 and Fig. 4). δ^13^C-FAs (C20–32) were relatively enriched in the fine fraction samples compared to the bulk in the case of MF (Table 1), but were, similar in the case of LL and UP. The δ^13^C-FAs exhibited a narrower range in the fine (−34.4 to −27.3‰) compared with the bulk (−37.3 to −28.7‰) samples for MF. In contrast, both bulk and fine samples exhibited similar δ^13^C-FA values in the case of UP and LL. δ^13^C values of C20 and C24 were more depleted in MF than UP (*p* < 0.05) in the fine fraction, while other FA isotopic composition remained similar across the land use sources (Table 1). δ^13^C-FA values of C20, C22, C24 and C26 differentiated MF and UP, while C28, C30 and C32 clearly distinguished MF and LL (*p* < 0.05). Similar to MF, a narrow range of δ^13^C values was observed in the fine (−34.6 to −29.8‰) compared to the bulk (−37.0 to −31.9‰) suspended sediment samples. δ^13^C-FA values for C20, C22, C24, C26, C28 and C30 were more depleted in the bulk compared to the fine fraction of the suspended sediment samples (*p* < 0.05) (Fig. 3), while the C32 content remained similar in both suspended sediment sample fractions (Fig. 4).

**Table 1:** Average (±standard deviation) isotopic composition of long-chain saturated FAs in the bulk (<2 mm) and fine (<0.063 mm) source soil samples (MF = mixed forest, UP = upland, LL = lowland). Different letters represent significant differences between land uses (Tukey's HSD test, *p* < 0.05) for each of fractions.

Tracers	δ^13^C (‰) value of fraction					
	Bulk fraction (<2 mm)			Fine fraction (<0.063 mm)		
	MF (n = 7)	UP (n = 6)	LL (n = 5)	MF (n = 7)	UP (n = 6)	LL (n = 5)
C20	−33.1 ± 2.1a	−29.9 ± 0.6b	−30.2 ± 1.1b	−31.6 ± 1.2a	−29.4 ± 0.6b	−30.9 ± 0.9ab
C22	−31.4 ± 1.6a	−29.5 ± 0.4b	−30 ± 0.3ab	−30.5 ± 1.1	−29.5 ± 0.5	−30.2 ± 0.4
C24	−30.5 ± 1.7a	−27.4 ± 0.7b	−28.7 ± 1.3ab	−29.3 ± 1.5a	−27.1 ± 0.3b	−28.1 ± 0.4ab
C26	−31.2 ± 1.8a	−28.6 ± 0.6b	−29.7 ± 1.1ab	−30.1 ± 1.9	−28.2 ± 0.6	−29.7 ± 1.1
C28	−32.4 ± 1.7a	−29.5 ± 1.1b	−28.8 ± 1.3b	−30.4 ± 1.9	−29.2 ± 0.2	−29.2 ± 1.1
C30	−33 ± 1.3a	−31 ± 1.4ab	−30.1 ± 1.7b	−31.4 ± 1.6	−30.4 ± 0.6	−30.4 ± 1.3
C32	−34.1 ± 2.1a	−31.5 ± 1.7ab	−29.9 ± 1.7b	−32.3 ± 1.8	−30.7 ± 1	−30.5 ± 1.4
Bulk	−25.6 ± 1.7a	−21.9 ± 0.9b	−23.3 ± 0.9ab	−25.9 ± 1.1a	−22.8 ± 0.7b	−23.8 ± 0.8b

### 3.3 Sediment source apportionment

**Fig. 5:**
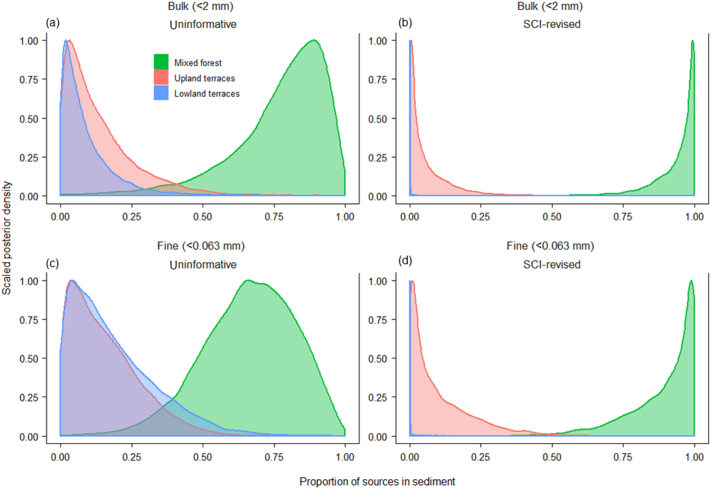
Sediment source apportionment using MixSIAR (a, c) without and (b, d) with informative priors for the (a, b) <2 mm and (c, d) <0.063 mm fractions of target sediment samples collected from the Kharka catchment.

Using the BIMM with uninformative priors (i.e., assumed equal connectivity for all sources) and data from bulk target sediment samples, the means and [95% CI] for the source contributions were 77% [33–97%], 14% [0–47%] and 9% [0–38%] for MF, UP and LL, respectively, compared to 66% [31–93%], 16% [0–43%] and 18% [0–53%], respectively, using data for the fine fraction only (Fig. 5b, d). When SCI-revised was used as an informative prior in the BIMM, the mean and [95% CI] for the contribution from MF changed to 94% [72–100%] when the bulk and to 90% [59–100%] when the fine fraction data were used (Fig. 5a, c). Interestingly, UP contributions appeared dominant compared to LL in both cases when the SCI-revised was used as an informative prior. In comparison, proportional contributions from the three land uses (Table 2, and Supplementary Table S3) to the target sediment samples were slightly changed compared to those obtained from the BIMM with uninformative priors when SCI-initial was used as an informative prior in the MixSIAR framework. Matrix plots showed strong negative correlation (−0.70) between MF and LL contributions for fine fraction (*p* < 0.001) but changed into weak correlation (−0.28) while SCI-revised was used as an informative prior (Fig. S5). Overall, source contribution estimates increased in precision in tandem with prior information becoming more informative (i.e. SCI-revised).

**Table 2:** Estimates of the proportional contribution of mixed forest, upland and lowland terraces to fine sediments in the Kharka catchment Nepal. Proportional contribution represents posterior density distributions of MixSIAR formulated with different priors.

Priors	Land use	Mean ± SD	95% CI	Bias	MSE (accuracy)	Precision
Uninformative	Mixed forest	0.66 ± 0.16	0.31–0.93	NA	NA	NA
SCI-initial	Mixed forest	0.70 ± 0.15	0.36–0.94	0.03	0.025	0.024
SCI-revised	Mixed forest	0.89 ± 0.11	0.59–1	0.235	0.068	0.012
Uninformative	Upland	0.16 ± 0.12	0.01–0.43	NA	NA	NA
SCI-initial	Upland	0.15 ± 0.12	0.01–0.44	0.004	0.015	0.015
SCI-revised	Upland	0.09 ± 0.10	0.01–0.38	−0.061	0.015	0.011
Uninformative	Lowland	0.18 ± 0.14	0.01–0.53	NA	NA	NA
SCI-initial	Lowland	0.15 ± 0.13	0.01–0.49	−0.035	0.020	0.019
SCI-revised	Lowland	0.01 ± 0.03	0.01–0.11	−0.17	0.032	0.002

## 4 Discussion

### 4.1 Importance of particle size in CSSI sediment source apportionment

PSDs differed between source soil and target sediment samples potentially due to hydro-dynamic processes. Several factors can influence the PSD of sediments including: 1) the PSD of the source soil; 2) aggregate breakdown due to erosion and turbulence, and; 3) the settling velocity of different particle size classes (Shi et al., 2012). The PSD of source soil is governed by the parent material, weathering and erosion processes. The similar PSD (Fig. S1) for LL (span i.e. difference between D_90_ and D_10_ = 0.063 mm) and target sediment (span = 0.061 mm) further suggests that cultivation practice (e.g. puddling) also affects the PSD of the soil. Minor differences in the δ^13^C-FA values between the bulk and fine fractions collected from UP and LL (Table 1) can be explained by the deposition of fine sediment depleted in ^13^C which originated mainly from MF. Most of the eroded soil from MF and UP is also transferred to the LL through natural run-on as well as through irrigation water and deposited on the LL terraces. Carver (1997) estimated sediment accumulation on the irrigated terraces in the middle-hills of Nepal at 6.6 mm y^−1^. The similarity between the chemically-dispersed PSD of LL terrace soil and target sediment (Fig. S1) further suggests that the δ^13^C-FA values in the LL soil samples are most likely influenced by sediment dynamics. Here, it is worth noting that suspended particles are not transported as chemically-dispersed particles, but rather as aggregated flocs regardless of the discharge velocity (Grangeon et al., 2012). Therefore, any similarity in the chemically-dispersed PSD did not provide reliable information on the sediment source contributions. Here, puddling operations for rice transplantation are likely to be an additional control on fining of the PSD. UP and LL terraces receive farmyard manure (FYM; Fig. 2b) made from the forest litter (depleted in ^13^C) which can increase long-chain FA content in FYM amended soil (Bull et al., 1998). Therefore, the effect of sediment accumulation and the addition of FYM with more negative δ^13^C values outweighed possible effects associated with carbon incorporation from maize crops into the surface soils of both UP and LL terraces.

The deposition and remobilisation of sediment along the Kharka catchment channel system is minimal because of steepness and high flow velocities. δ^13^C-FA values were relatively depleted (by 4‰) in the bulk compared to fine sediment samples, which is likely due to the influence of fresh and/or partly decomposed organic detritus (Chikaraishi and Naraoka, 2006). Headwater streams are expected to have a high proportion of organic matter in their sediment (McCorkle et al., 2016). The mass of the 0.063–2 mm fraction in the target sediment samples was <20%. We did not analyse this fraction; however, the predominant input of plant-derived saturated long-chain FAs has previously been reported for free particulate organic matter (Wiesenberg et al., 2010) and the >0.063 mm fraction (Angst et al., 2018). Consequently, δ^13^C values (except C20, C28 and C30) of the <2 mm fraction of target sediment was found to be outside the range of potential source soils (Fig. S3). In contrast, δ^13^C values for fine sediment were found to lie within the range of soil samples from MF, UP and LL. Apart from the effect of organic matter enrichment on the isotopic composition of bulk sediment samples, stochastic erosion processes in high mountainous elevations can also induce high variability in signals (Smith et al., 2013) that might not be captured properly during source soil sampling. In essence, source soil samples sieved based on the dominant PSD of target sediment samples (in this study i.e. <0.063 mm) could exclude the influence of both fresh and/or partially decomposed organic matter and grain size variation in the estimation of source contributions. Abrasion of fine particles (<0.063 mm) during transport is unlikely since kinetic energy transfer during collision is minimal (Jerolmack and Brzinski, 2010) and, therefore, less likely to provide the surface area for re-adsorption of desorbed FAs. The influence of this process on the sediment source apportionment is difficult to quantify; however, it is assumed that most of the eroded materials are carried downstream in the high energy study catchment. Whether the abrasion affects FAs adsorption and desorption during transport is currently unknown. Regardless, the observed pattern of δ^13^C-FA values in source soil and target sediment samples supports the necessity of tracer analysis in fractions based on the sediment PSD since FA content is sensitive to changes in particle size and organic matter content.

### 4.2 Linkages between the sediment contribution from community-managed forest and topography and management

Run-off and sediment redistribution in catchments is significantly influenced by delivery pathways i.e. sediment/hydrological connectivity. The SCI-revised values for different land uses (Fig. 3b) showed that the MF on hillslopes is highly connected to the channel network compared to UP and LL terraces. The SCI-revised consists of several variables (e.g. catchment configuration, man-made structures, ground coverage, etc.) well-established conceptually to control the spatial configuration and intensity of sediment fluxes in a catchment (Cavalli et al., 2019; Heckmann et al., 2018). The high sediment contributions from MF (Fig. 5, Table 2) in the Kharka catchment suggested that steep gradient (Fig. 1b) as well as high drainage density support sediment transmission from the steep hillslopes to the stream which is consistent with Fryirs' (2013) conceptualization of the sediment cascade. Strong hillslope-to-channel and channel-to-valley floor connectivity are the main pathways enhancing sediment delivery from MF. Reduction of surface coverage due to litter collection (Fig. 2a) can enhance runoff connectivity (Mayor et al., 2008), which might exacerbate soil erosion in forest. We do not have proper data about the extent of ground cover removed from the forest, but soil erosion is generally severe (Fig. S6) if the surface coverage falls below 50% (Feng et al., 2016). Densely distributed low-order streams on steep slopes (Fig. 1a, b) effectively couple hillslopes to the channel network (Marteau et al., 2017) to deliver eroded materials. The higher the drainage density, the shorter the runoff distance and sediment travel over the sloping surface before entering a channel (Kirkby et al., 2002). Additionally, hillslope-to-channel connectivity generally depends on slope gradient, slope length and vegetation type and ground coverage which are all captured well by the SCI-revised. Energy for erosion and sediment transport is directly proportional to slope gradient. The slope gradient increases in the downslope direction in MF meaning that erosion and sediment transport potential also increases. Indeed, steeper slopes have less surface storage mainly due to the effective drainage of depressions that would otherwise act as sinks on gentle slopes. The differences between the SCI-initial and SCI-revised values (Figs. 3, S2) also highlighted the influence of the land cover and practice in the estimation of the SCI. It should be noted that sediment connectivity is dynamic in nature and varies temporarily based on land cover changes due to agricultural and forest management practices. Additionally, connectivity also changes with the magnitude of hydrologic events. There is, therefore, great benefit in incorporating factors like land cover and management (i.e., SCI-revised) since the influence of these on connectivity cannot be detected by an index solely derived from topography (i.e., SCI-initial). Land cover change and management practices such as terracing affect sediment connectivity by influencing runoff and sediment generation, routing and dynamics (Heckmann et al., 2018).

As the study stream exits from the forest, a notable change in the hydrological network is observed in the field due to the construction of small canals to irrigate LL terraces. Sediment is deposited on the lower terraces that originates from upper terraces throughout this whole toposequence. Irrigation canals and terracing slow down sediment transfer (Fryirs, 2013) and result in sediment accumulation on the valley floor and LL terraces, thereby creating a local net sediment sink (Brown et al., 1999). Incorporation of SCI-revised in the BIMM as an informative prior helps capture this observable impact since only a nominal contribution was estimated for the agriculture terraces (Fig. 5). Without inclusion of the SCI-revised, the source apportionment cannot capture the combined influence derived from topographic features, land cover and practices and the resulting very low sediment contributions from UP and LL agricultural land (Fig. 5). Consequently, the posteriors predicted by the BIMM including SCI-initial make less environmental sense in the context of on-the-ground visual observations. The decreased DIC (from 42.2 to 40.7 in the case of the fine fraction and from 21.24 to 17 in the case of the bulk fraction) due to the use of SCI-revised as prior information indicates a better model fit and more accurate point estimates. Overall, substantial variability and uncertainty in the estimated source contributions to target sediment were observed due to the similarity of the isotopic tracers between the individual land uses (Table 1, Fig. S5). Inclusion of SCI-revised in the BIMM increased the negative correlation between MF and UP posterior contributions (Fig. S5b) compared to model with uninformative priors indicating that priors have influenced in these sources contribution. It should be noted that negative correlation between posteriors indicates the low ability of the CSSI tracers to discriminate between the individual sources. Therefore, strong correlation values of source proportions calculated after incorporation of informative priors do not suggest that sources are close to each other in terms of tracer values.

Incorporation of SCI-revised as an informative prior in the BIMM improved the precision of our posteriors more than using SCI-initial, and without systematically biasing the uninformative model estimates (Table 2). This incremental improvement in precision suggests that retaining the posteriors based on the uninformative priors (i.e. assumed equal connectivity for all sources) is inappropriate (Lemoine, 2019; Van Dongen, 2006). These results thereby suggest that inclusion of appropriate priors can improve mixing model inference (Phillips et al., 2014; Stock et al., 2018; Ward et al., 2010). Incorporation of at least some prior knowledge in BIMM has been suggested in ecology but, to date, this approach has been highly underutilised in sediment source apportionment studies. Our study showed that sediment source estimation can benefit from appropriately formulated priors and we encourage end-users of sediment fingerprinting to explore incorporation of SCI as prior information in BIMM. There is currently very little quantitative information on the influence of irrigation canals and terraces on sediment dynamics at the catchment scale in the study area. Therefore, it is difficult to validate the sediment source apportionment results of this study independently but, the comparison of the posteriors generated by the different versions of the BIMM suggest incremental changes in conjunction with the inclusion of SCI-revised rather than SCI-initial and the final predicted source proportions make sound environmental sense in the context of prior quantitative knowledge concerning the net soil loss from forests (up to 4.48 kg m^−^^2^) vs bench terraces (1% slope; up to 0.01 kg m^−^^2^) (Morgan, 2001), qualitative understanding of forest erosion (ICIMOD, 2007) and visual evidence in the study catchment. The need to improve sediment source information in this context has been stressed previously (Fu et al., 2013) and indeed used as a means of verifying predicted source proportions in the absence of independent scientific monitoring data (Slattery et al., 1995, Slattery et al., 2000; Walden et al., 1997). Clearly, the development of a sediment budget at multiple scales could be used to complement the CSSI fingerprinting study reported here, and to confirm scientifically, the robustness of the source apportionment data using SCI-revised, but such an approach would be resource intensive. This constraint is common and leads to the integration of source fingerprinting and additional line-of-evidence e.g. sediment connectivity, soil loss information and visual evidence as a pragmatic basis for assessing BIMM predictions.

The estimates of disproportionately high-sediment contributions from the upslope MF relative to UP and LL terraces might also suggest the potential impact of forest fires and earthquakes in the mid-hill region of Nepal. We do not have information on the incidence and severity of forest fire in the catchment but large earthquakes (e.g. the 2015 Gorkha Nepal earthquake) contribute significantly to sediment export from mountain forests (Frith et al., 2018). Sustainable management of community forest is critical for ensuring continued flow of goods and services for sustaining the livelihoods of smallholder farming communities across the mid-hills region of Nepal. Farmers prefer livestock to crops as a supplement to household incomes and thereby depend significantly on forest resources since livestock grazing and litter collection are widespread practices in the forest of the Kharka catchment and both increase vulnerability to soil erosion by decreasing surface roughness and sediment buffering (Fryirs and Brierley, 1999; Gardner and Gerrard, 2002; Li et al., 2014). Our study challenges the traditional perception that the vulnerability of agricultural land to erosion means it is the major source of sediment in the mountainous terraced catchments. Previous studies by Upadhayay et al. (2018b), Brown et al. (1999) and ICIMOD (2007) in the region have also reported that hillslope forests are highly reactive to rainfall and management in terms of sediment generation while the terraced agriculture systems are comparatively sluggish. Sediment source apportionment results are, however, highly catchment specific and further research is necessary to understand the regional impact of terracing on sediment dynamics and corresponding downstream sediment source apportionment.

## 5 Conclusions

Over recent decades, research on sediment fingerprinting and connectivity has advanced in parallel but independently, underscoring the scope for integration of these approaches. This paper is the first to explore the suitability of a SCI as important prior information in sediment source apportionment using a BIMM for supporting evidence-based conservation strategies under changing climate and forest management practices. Sediment source apportionment using a SCI as an informative prior indicated that high sediment connectivity created by both topography and over-exploitation of forest resources results in high sediment contributions to the catchment outlet. Specifically, our results indicate that sediment fingerprinting modelling requires directly comparable source and sediment particle size classes to account for variation of FAs content and associated δ^13^C values of sediment owing to particle size fining during erosion, transport and deposition. Management interventions should be specifically targeted to grazing and litter management in the community forest for soil erosion control in the study area.

## Declaration of competing interest

The authors declare that there is no conflict of interest.

## Appendix A Supplementary data

Supplementary material Image 1
