# Supplementary material for: Sensitivity of source apportionment predicted by a Bayesian tracer mixing model to the inclusion of a sediment connectivity index as an informative prior: Illustration using the Kharka catchment (Nepal)
Source: Sci Total Environ. 2020 Apr 15;713:136703. doi: 10.1016/j.scitotenv.2020.136703 (PMC7043003; doi:10.1016/j.scitotenv.2020.136703)
Supplement: Supplementary file 1 — Supplementary material [file mmc1.docx]

Supplemental Information

**Sensitivity of source apportionment predicted by a Bayesian tracer mixing model to the inclusion of a sediment connectivity index as an informative prior: illustration using the Kharka catchment (Nepal)**

Hari Ram Upadhayay^1,6*^, Sushil Lamichhane^2,3^, Roshan Man Bajracharya^4^, Wim Cornelis^5^, Adrian L. Collins^1^, Pascal Boeckx^6^

*^1^Sustainable Agriculture Sciences, Rothamsted Research, North Wyke, Okehampton, EX20 2SB UK.*

*^2^School of Environmental and Rural Science, University of New England, Armidale, Australia*

*^3^Soil Science Division, Nepal Agricultural Research Council, Nepal*

*^4^Department of Environmental Science and Engineering, Kathmandu University, Nepal*

*^5^Soil Physics (SoPHY), Ghent University, Coupure Links 653, 9000, Gent, Belgium*

*^6^Isotope Bioscience laboratory-ISOFYS, Ghent University, Coupure Links 653, 9000, Gent, Belgium*

| 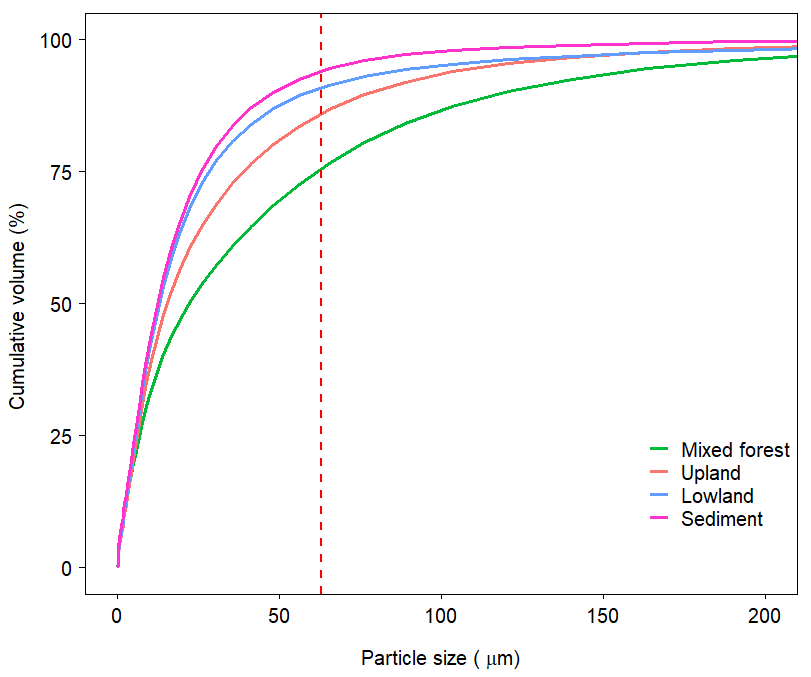  **Figure S1** Average cumulative particle size distributions for source and suspended sediment samples collected in the Kharka catchment. Vertical dotted red line respresents the D_90_ of the target sediment samples. |
| --- |

|   **Figure S2** Sediment connectivity indices (SCI-initial and SCI-revised) of each land use type (mixed forest, n=16562; upland, n=16059; lowland, n=3038) for the Kharka catchment, Nepal. Horizontal bold line and open circle in the box indicate median and mean values respectively. Boxes represent interquartile ranges (IQR), whiskers represent 1.5 times IQR and individual dots beyond the whiskers indicate outliers. |
| --- |

| 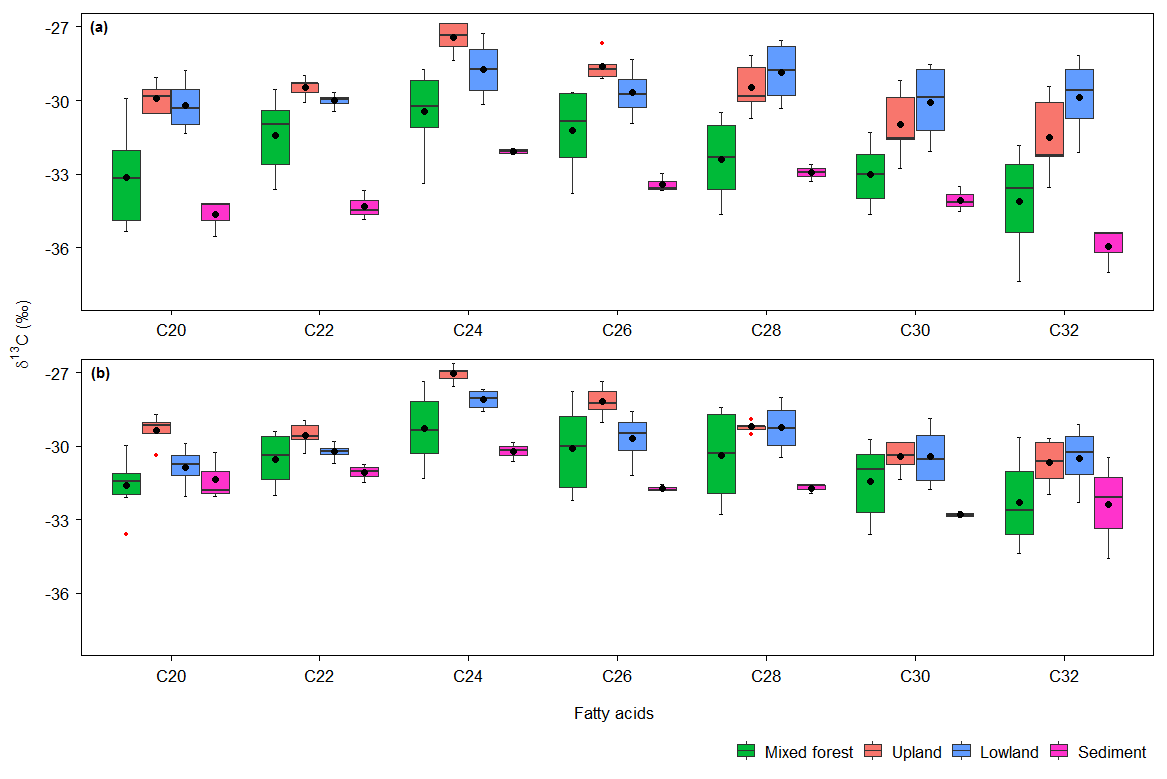  **Figure S3** δ^13^C values of fatty acids in the (a) bulk (<2 mm) and (b) fine (<0.063 mm) fractions of source soil (mixed forest, n=7; upland, n=6; lowland, n=5) and target sediment (n=9) samples. Horizontal line and closed circle in the box indicate median and mean values respectively. Boxes represent interquartile ranges (IQR), whiskers represent 1.5 times IQR and individual dots beyond the whiskers indicate outliers. |
| --- |

| 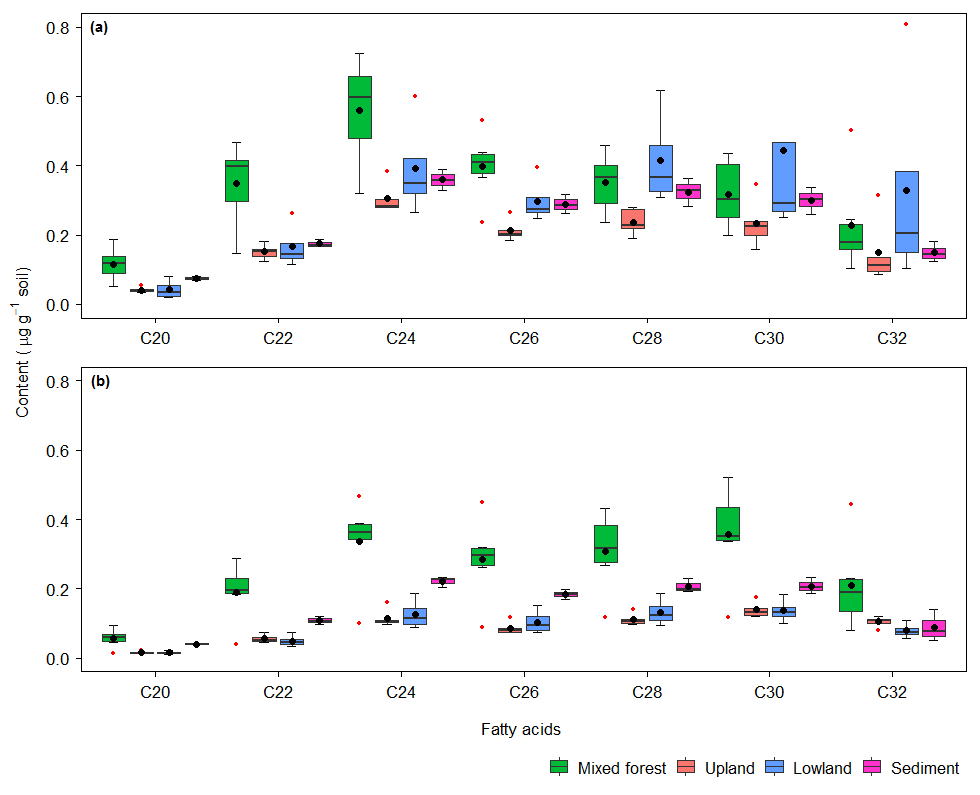  **Figure S4** Fatty acids content in the (a) bulk (<2 mm) and (b) fine (<0.063 mm) fractions of source soil (mixed forest, n=7; upland, n=6; lowland, n=5) and target sediment (n =9) samples. Horizontal line and closed circle in the box indicate median and mean values respectively. Boxes represent interquartile ranges (IQR), whiskers represent 1.5 times IQR and individual dots beyond the whiskers indicate outliers. |
| --- |

|   (a) |
| --- |
|   (b) |
| **Figure S5** Matrix plot showing correlations between the posteriors source (MF, mixed forest; UP, upland terraces; LL lowland terraces) contributions to fine target sediment estimated using (a) uninformative priors and (b) SCI-revised as an informative prior in MixSIAR. Contour plots in the upper diagonal show the relationship bewetten the sources , histograms on the diagonal represents source proportion, correlation coefficients between sources (***p<0.001) in the lower diagnol. |


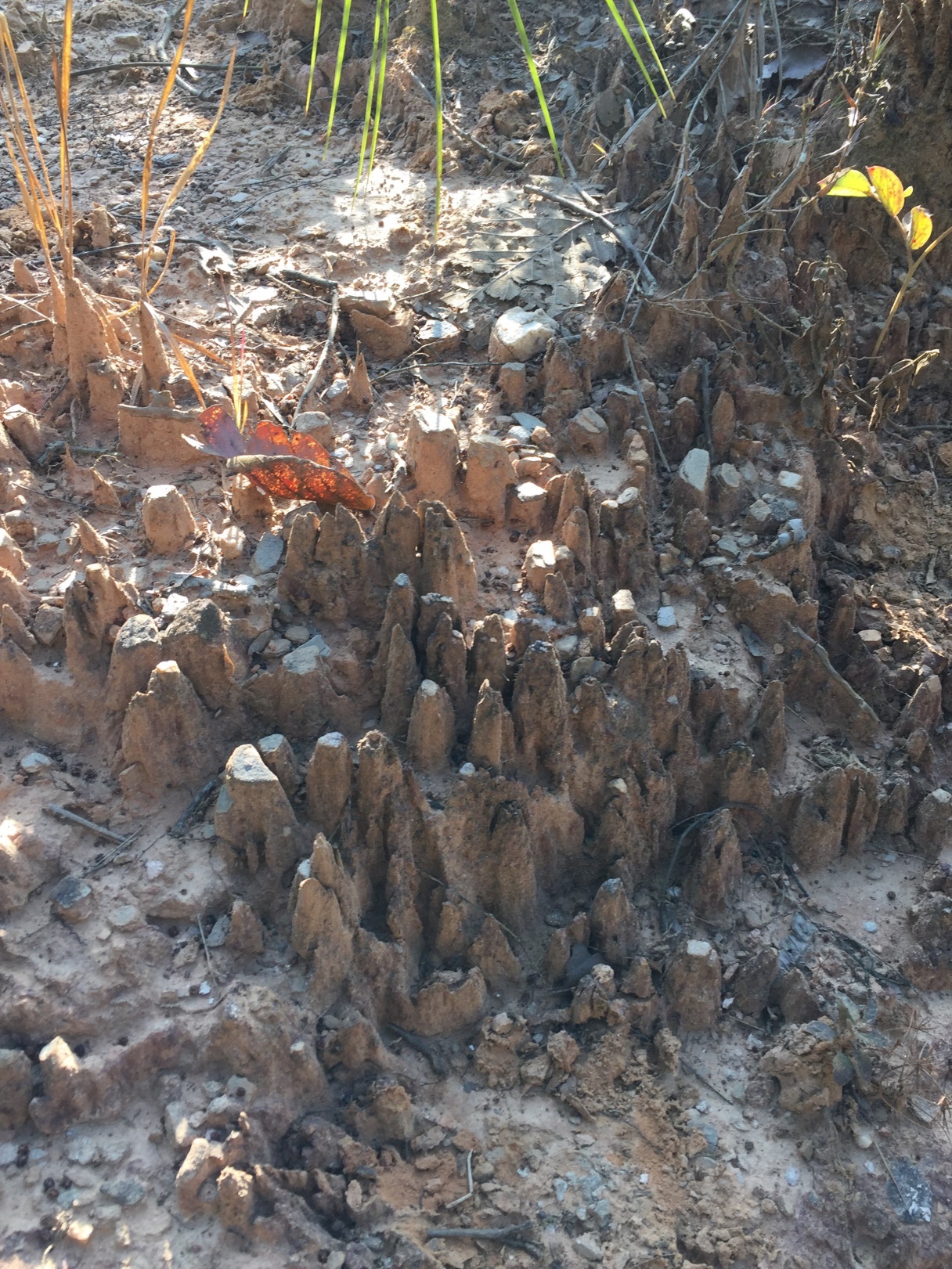


**Figure S6** Lack of effective ground cover effect on soil erosion under the tree.

Table S1 Summary particle size characteristics (volume % basis) of the source material and target sediment samples.

| Sources and sediment | Clay (<0.02mm) | Silt (0.02-0.063mm) | Sand (>0.063mm) |
| --- | --- | --- | --- |
| Mixed forest | 47 | 28 | 25 |
| Upland | 57 | 27 | 16 |
| Lowland | 64 | 26 | 10 |
| Target sediment | 65 | 29 | 06 |

Table S2 Estimation of the weighing factor based on the land cover and practices in the Kharka catchment (detail in Chapter 5 in Renard et al. 1997 for description of each variable and corresponding possible values).

| Land use | Variables | | | | | Cover | | ^†††^Practice (P) | Overall weighing factor  (CC×SC×P) |
| --- | --- | --- | --- | --- | --- | --- | --- | --- | --- |
|  | ^†^F_c_ | H | b | ^††^S_p_ | R_u_ | Canopy cover sub-factor (CC) | Surface cover sub-factor (SC) |  |  |
| Forest | 0.7 | 8 | 0.039 | 55 | 4 | 0.69 | 0.18 | 1 | 0.12 |
| Upland | 0.5 | 2 | 0.035 | 30 | 0.24 | 0.59 | 0.35 | 0.14 | 0.03 |
| Lowland | 0.5 | 2 | 0.035 | 30 | 0.24 | 0.59 | 0.35 | 0.14 | 0.03 |

^†^Overall canopy cover from April to November based on author and farmer experience. Tree density decreases with increasing altitude. Mixed forest and agriculture terraces have low canopy cover in April but high cover in November. Mixed forest is deciduous.

^††^Overall surface cover from April to November. Note that pines have restricted growth of understory vegetation and farmers remove leaf litter layer from forest. In April there is almost no surface cover on agricultural terraces and surface cover increases with the onset of the monsoon.

^†††^ Upland and lowland have levelled bench terraces while mixed forest has no erosion control practices.

where, F_c_= fraction of land surface cover, H= canopy height (ft), b= empirical coefficient (see Renard et al. 1997), S_p_= percentage of land area covered by surface cover, R_u_=surface roughness.

Table S3 Mean (± SD) posterior distributions estimated from Bayesian isotope mixing models for the proportional contributions of the three land uses to target sediment from the outlet of the Kharka catchment, Nepal.

| Priors | Sediment type | MF | UP | LL |
| --- | --- | --- | --- | --- |
| N | Fine | 0.66 ± 0.16 | 0.16 ± 0.12 | 0.18 ± 0.14 |
| SCI-initial | Fine | 0.69 ± 0.15 | 0.15 ± 0.12 | 0.14 ± 0.13 |
| SCI-revised | Fine | 0.89 ± 0.11 | 0.10 ± 0.10 | 0.01 ± 0.03 |
| N | Bulk | 0.77 ± 0.16 | 0.14 ± 0.12 | 0.09 ± 0.10 |
| SCI-initial | Bulk | 0.79 ± 0.14 | 0.13 ± 0.12 | 0.08 ± 0.08 |
| SCI-revised | Bulk | 0.94 ± 0.07 | 0.05 ± 0.07 | 0.01 ± 0.01 |

Three models were specified with three prior distributions (i.e. uninformative [N], an informative prior estimated based on topographic parameters [SCI-initial] and an informative prior estimated based on topography, landcover and practices [SCI-revised]) for two target sediment types i.e. fine (<0.063mm) and bulk (<2mm) fractions.

Refereces

Renard KG, Foster GR, Wessies GA, McCool DK, Yoder DC. Predicting Soil Erosion by Water: A Guide to Conservation Planning with the Revised Universal Soil Loss Equation (RUSLE). Agriculture Handbook , vol. 703: U.S. Department of Agriculture, Agricultural Research Service, 1997.
